# Radiographic Analysis of Bone Remodelling Around a Novel 3D-Printed, Highly Porous Titanium Monobloc Dual-Mobility Cup in Total Hip Arthroplasty at 2-Year Follow-Up

**DOI:** 10.3390/biomimetics11100693

**Published:** 2026-09-28

**Authors:** Nicole Puteo, Cesare Donadono, Matteo Cuzzani, Maria Antonietta Coppola, Claudia Rondinella, Domenico Tigani

**Affiliations:** Department of Orthopaedic Surgery, Ospedale Maggiore Carlo Alberto Pizzardi, 40133 Bologna, Italy

**Keywords:** total hip arthroplasty, dual-mobility cup, 3D printing, highly porous, bone remodelling, titanium, osseointegration

## Abstract

Dual-mobility cups (DMCs) are traditionally manufactured from stainless steel or cobalt–chromium, materials with substantially greater stiffness than cancellous bone that may contribute to stress shielding and periacetabular bone resorption. A recently introduced 3D-printed titanium alloy DMC features a highly porous randomized trabecular architecture at the bone–implant interface. This study aimed to compare the short-term radiographic osseointegration and changes in periacetabular relative bone density (RBD) of this novel titanium DMC with those of conventional third-generation DMCs. A total of 204 patients who underwent cementless total hip arthroplasty were retrospectively included. Propensity score matching was used to identify two comparable groups: Titanium (*n* = 88) and Conventional (*n* = 88). Radiographic assessment at a minimum of 2-year follow-up included Moore’s signs of osseointegration and the presence of radiolucent lines (RLLs). Relative bone density was measured in three regions of interest (ROIs) around the cup using ImageJ, and percentage changes from the postoperative assessment to the 2-year follow-up were calculated. Both groups demonstrated satisfactory radiographic fixation, with at least three Moore signs present in 100% of the Titanium group and 97.7% of the Conventional group (*p* = 0.497). No RLLs were observed in the Titanium group at final follow-up, whereas six hips (6.8%) in the Conventional group exhibited RLLs > 2 mm (*p* = 0.029). Both groups showed decreases in RBD across all ROIs, with no statistically significant between-group differences. At a minimum of 2-year follow-up, the novel 3D-printed titanium DMC demonstrated satisfactory radiographic fixation, with no statistically significant differences in periacetabular RBD compared with the heterogeneous group of conventional DMCs. These preliminary short-term findings require confirmation in prospective studies incorporating validated clinical outcomes, quantitative imaging, and longer follow-up.

## 1. Introduction

Dual-mobility cups (DMCs) were pioneered in France in the late 1970s to reduce wear and dislocation after total hip arthroplasty (THA) [1,2]. Since then, monobloc DMCs have evolved in design and fixation interface, although their shell materials have remained largely unchanged. DMCs are commonly made of stainless steel (SS) or cobalt–chromium (CoCr) alloy because of their favourable tribological properties when articulating with polyethylene [3]. Notably, the elastic moduli of CoCr alloy and SS (210–240 GPa) are approximately twice that of titanium alloys (110 GPa) and two orders of magnitude greater than that of cancellous pelvic bone (0.1–2 GPa) [4,5]. This stiffness mismatch between implant and bone may lead to stress shielding of the adjacent bone, thereby promoting localized bone resorption and potentially contributing to implant loosening [6]. In a recent randomized controlled trial, higher periacetabular bone mineral density (BMD) was observed around a polyethylene cup with a plasma-sprayed titanium coating than around a modular titanium-coated cup [7]. The polyethylene cup, which has an elastic modulus of approximately 1 GPa, was associated with significantly less bone loss in the polar region, although both cups were well osseointegrated.

Several studies have compared the osseointegration of highly porous standard acetabular cups with hydroxyapatite-coated titanium modular cups [5,8], reporting higher BMD around highly porous cups at 10-year follow-up. Increased porosity and trabecular-like geometry may enable deeper and more uniformly distributed bone ingrowth than conventional porous coatings [9]. In 2021, a monobloc DMC featuring a bioinspired bone-facing architecture was introduced. The ACORN TRASER cup is manufactured entirely as a monobloc Ti6Al4V (titanium–6aluminum–4vanadium) alloy component by three-dimensional printing using selective laser melting. According to the manufacturer’s technical documentation, its bone-facing surface has 70% porosity, a mean pore size of 520 μm, and an irregular trabecular architecture generated using a Voronoi pattern. This interconnected porous structure is intended to reproduce key structural features of cancellous bone, provide pathways for osteoblast colonization, and support bone ingrowth. The bearing surface is coated by physical vapour deposition (PVD) with titanium-niobium nitride (TiNbN). The trabecular portion covers the bone–implant interface, and an elastic modulus of approximately 1.5 GPa has been reported in the cited literature [10,11], closer to cancellous bone than bulk metallic alloys. More broadly, previous studies have described selective-laser-melting design and process optimization for patient-specific bone implants and the mechanical characterization of additively manufactured orthopedic alloy components, providing additional technological context for porous joint implant development [12,13].

To our knowledge, periacetabular bone remodelling around DMCs made from different materials remains insufficiently investigated. It is unknown whether a highly porous titanium DMC produces different periprosthetic bone remodelling from conventional DMCs. The purpose of this study was to compare this titanium DMC with conventional third-generation DMCs in terms of radiographic osseointegration signs and changes in relative bone density (RBD) at a minimum 2-year follow-up. We hypothesized that the lower stiffness of the titanium construct might be associated with reduced periacetabular RBD loss; this hypothesis was considered exploratory.

## 2. Materials and Methods

### 2.1. Study Design and Patient Selection

This retrospective comparative study included patients treated between January 2022 and December 2023. During this period, 382 patients (391 hips) underwent cementless THA with either titanium or conventional DMCs at the same tertiary-care hospital. Patients with titanium or conventional monobloc DMCs and anteroposterior (AP) radiographs obtained at a minimum of 2-year follow-up were eligible. The exclusion criteria were a modular DMC, revision surgery, documented or suspected infection, and the presence of screws or cages in the acetabulum. After screening, 204 patients (204 hips) were included. Patients who received the titanium DMC (*n* = 101) comprised the investigational group (Titanium group), whereas those who received a conventional DMC (*n* = 103) comprised the control group (Conventional group). To reduce selection bias and obtain comparable groups, propensity score matching (PSM) was performed using age, sex, diagnosis, and follow-up duration. After PSM, 88 patients (88 hips) were included in each group (Figure 1).

### 2.2. Surgery and Implants

All procedures were performed by the same senior surgeon through a posterolateral approach, as described by Moore, with short external rotator detachment, piriformis and joint-capsule repair, and transosseous fixation [14]. From the first postoperative day, patients were allowed full weight-bearing with assistive devices as needed. Radiographic and clinical evaluations were performed postoperatively, at 1, 6, and 12 months, and annually thereafter.

Patients in the Titanium group received the ACORN TRASER cup (Permedica Orthopaedics, Merate, Italy), a monobloc DMC manufactured entirely from Ti6Al4V titanium alloy using three-dimensional printing by selective laser melting. Its bone-facing surface has 70% porosity, a manufacturer-reported mean pore size of 520 μm, and an irregular trabecular structure generated using a Voronoi pattern. The bearing surface is coated by PVD with TiNbN. Patients in the Conventional group received heterogeneous third-generation monobloc DMCs, including Novae Sunfit TH (Serf, Lyon, France), ACORN Primary (Permedica Orthopaedics, Merate, Italy), X.CUP (X.NOV, Héricourt, France), Quattro (Groupe Lépine, Genay, France), and conventional cups manufactured by Adler and Intrauma. Despite an additional review of the available records, the exact numerical distribution among the conventional designs could not be reliably reconstructed.

### 2.3. Radiographic Assessment

Postoperative and last available follow-up AP pelvic radiographs, obtained at a minimum of 2 years, were retrieved from the hospital Picture Archiving and Communication System (PACS). All examinations were performed in the same radiology department. The images were anonymized, and two independent orthopedic surgeons assessed Moore’s radiographic signs of osseointegration and radiolucent lines (RLLs) without access to patient identifiers or clinical information [15]. Complete blinding to implant type and manufacturer could not be guaranteed because differences in implant geometry may have been recognizable radiographically. A cup was classified as radiographically osseointegrated when at least three Moore signs were present: (1) absence of radiolucent lines; (2) superolateral buttress; (3) medial stress shielding; (4) radial trabeculae; and (5) inferomedial buttress (Figure 2). RLLs wider than 2 mm in at least one DeLee and Charnley zone were recorded as present [16,17]. Disagreements regarding Moore signs and RLLs were resolved by consensus with the senior surgeon.

### 2.4. Relative Bone Density (RBD)

To assess bone remodelling without additional diagnostic imaging, we used routine radiographs and ImageJ 2.0 (Wayne Rasband, National Institutes of Health, USA) [18]. This grayscale-derived RBD is a relative surrogate and should not be considered equivalent to BMD measured by dual-energy X-ray absorptiometry (DEXA). Building on related radiographic density measurement methods [19], the measurements followed the algorithm described by Godoy et al. [20]. Postoperative and 2-year AP radiographs were converted to 8-bit grayscale. High and low thresholds were identified using the femoral head and the space outside the body, respectively, to adjust image brightness, and the mean grayscale value of the femoral head was recorded. Three pelvic regions of interest (ROIs) were then selected around the cup while avoiding superimposed dense structures (Figure 3). ROI 1 was a 10 mm high rectangle in the ilium immediately above the cup, avoiding the ilioischial line. ROIs 2 and 3 were 5 mm squares in the pubis and ischium, respectively. ROI 2 was positioned medial to the cup without including the ilioischial line or teardrop, and ROI 3 was positioned below the cup. Mean grayscale values were measured within each ROI and normalized to the mean grayscale value of the femoral head according to the following formula. Both observers performed the RBD measurements independently once; formal ICC analysis of the continuous measurements and repeated intra-observer measurements were not available.

RBD = (mean grayscale value in the ROI)/(mean grayscale value of the femoral head).

To assess changes in RBD, postoperative and 2-year follow-up values were calculated for each ROI. The percentage change (Δ%) was then calculated as follows:

(RBD at 2 years − postoperative RBD)/(postoperative RBD) × 100.

### 2.5. Statistical Analysis

PSM was used to reduce differences in measured baseline covariates. Patients were matched 1:1 by identifying pairs with the smallest average absolute distance within a calliper of 0.1 × σ [21]. Gender, age, diagnosis and follow-up were selected because they were consistently available in the retrospective dataset. Other potential confounders, including BMI, osteoporosis, smoking, diabetes, medication use, activity level, previous hip surgery and cup size, were not systematically available and could not be included. SMDs were calculated for all matching variables, with values >0.2 considered indicative of imbalance [21]. Inter-observer reliability for categorical Moore signs and RLLs was evaluated using Cohen’s kappa (κ) [22]. Formal inter-observer ICCs for continuous RBD measurements and intra-observer reliability were not calculated. Categorical variables were summarized as counts and percentages and compared using Fisher’s exact or chi-square tests. Continuous variables were summarized using means, standard deviations and ranges. Normality was evaluated using the Shapiro–Wilk test; Student’s t-test and the Mann–Whitney test were used for normally and non-normally distributed outcomes, respectively. Statistical significance was set at *p* ≤ 0.05. Because several radiographic outcomes were explored without correction for multiple testing, nominally significant findings should be interpreted cautiously. No a priori sample-size calculation was performed because the retrospective sample was determined by the available eligible cohort. An approximate sensitivity analysis for two independent groups (*n* = 88 per group), using a two-sided α of 0.05, 80% power, and the observed pooled standard deviations, indicated minimum detectable between-group differences of 6.2, 8.0, and 8.6 percentage points for Δ% RBD in ROIs 1, 2, and 3, respectively. XLSTAT Premium (Addinsoft, New York, NY, USA) was used for PSM, JASP version 0.95 (JASP Team, 2025) for categorical interrater reliability, and GraphPad Prism version 10 (GraphPad Software, Boston, MA, USA) for the remaining analyses.

## 3. Results

### 3.1. Patient Demographics

Among the 204 patients included in the study, 143 were female (70.0%) and 61 were male (30.0%). The mean age was 75.2 ± 10.1 years (range, 28–89 years). The most frequent indication for THA was femoral neck fracture (172 patients, 84.3%), followed by osteoarthritis (26 patients, 12.7%), osteonecrosis of the femoral head (four patients, 2.0%), pathological femoral neck fracture (one patient, 0.5%), and pseudoarthrosis (one patient, 0.5%). The mean follow-up was 2.5 ± 0.4 years (range, 2.0–3.8 years). Before PSM, the groups showed imbalances in sex and follow-up duration, with SMDs of 0.223 and 0.334, respectively, and a significant difference in follow-up duration (*p* = 0.029). After PSM, 88 patients (88 hips) were included in each group, with no significant differences or relevant imbalances in the matching variables. In both groups, the mean age was approximately 75 years, the most frequent indication was femoral neck fracture (77 patients, 87.5%), and the mean follow-up was 2.5 years (Table 1).

### 3.2. Radiographic Outcomes

At least three Moore signs were observed in 88 hips (100%) in the Titanium group and 86 hips (97.7%) in the Conventional group (*p* = 0.497), indicating satisfactory short-term radiographic fixation in both groups. Agreement varied across individual Moore signs (Table 2); a low κ despite high percentage agreement for infrequent findings may reflect prevalence imbalance.

After consensus resolution of all disagreements, no RLLs wider than 2 mm were identified in the Titanium group, whereas six hips (6.8%) in the Conventional group had at least one RLL wider than 2 mm (*p* = 0.029). The comparison was based on 0/88 versus 6/88 hips. These RLLs were non-progressive and asymptomatic and occurred in DeLee and Charnley zone I in four cups and zone III in two cups. During radiographic assessment, 29 hips (32.9%) in the Titanium group showed a postoperative gap adjacent to the equatorial rim in zone I. These gaps may have been related to equatorial over-reaming, although this mechanism could not be demonstrated retrospectively, and they had completely disappeared radiographically at final follow-up. This finding represents radiographic filling and does not provide direct histological evidence of bone ingrowth (Figure 4).

### 3.3. Relative Bone Density Outcomes

Across the three ROIs, the group mean RBD decrease ranged from 4.99% to 17.16% at 2-year follow-up. Mean decreases were numerically larger in the Conventional group in each ROI; however, no statistically significant intergroup differences were detected (Figure 5).

In both groups, the largest mean RBD decrease occurred in ROI 3 and the smallest in ROI 1. In ROI 1, mean change was −4.99% in the Titanium group and −8.65% in the Conventional group (*p* = 0.218). In ROI 2, mean change was −5.89% and −10.85%, respectively (*p* = 0.160). In ROI 3, mean change was −11.96% and −17.16%, respectively (*p* = 0.138) (Table 3). The observed absolute between-group differences (3.66, 4.96, and 5.20 percentage points in ROIs 1, 2, and 3, respectively) were smaller than the corresponding minimum detectable differences estimated by the sensitivity analysis. These non-significant numerical differences should therefore not be interpreted as evidence of superior bone preservation, and modest between-group effects cannot be excluded.

## 4. Discussion

To our knowledge, this is the first short-term radiographic study of bone remodelling around a highly porous titanium monobloc DMC. The investigated cup incorporates a 3D-printed randomized trabecular structure at the bone–implant interface [10,11]. We evaluated radiographic fixation and periacetabular remodelling using Moore signs and grayscale-derived RBD, respectively, and compared one specific titanium design with a heterogeneous group of conventional DMCs.

Both groups demonstrated satisfactory short-term radiographic fixation. All cups in the Titanium group and 86 cups (97.7%) in the Conventional group showed at least three Moore signs. RLLs wider than 2 mm were identified in six Conventional hips and none in the Titanium group; however, these lines were non-progressive and asymptomatic, and their long-term clinical relevance remains uncertain. RBD decreased across all ROIs in both groups, and none of the intergroup comparisons reached statistical significance. The observed numerical differences are exploratory and do not establish superior bone preservation, biological fixation or clinical performance of the titanium implant.

The use of DMCs has increased over the past decade, not only among patients at high risk of dislocation but also among those with high functional demands [23,24]. In patients with osteoarthritis, DMCs have overall revision rates comparable to those of acetabular cups with fixed liners, while reducing the risk of revision specifically due to dislocation by approximately 50% [25]. Contemporary conventional DMC designs have shown 100% survivorship free from acetabular aseptic loosening and excellent osseointegration at follow-up of up to 10 years [25,26,27,28]. Moreover, DMCs have proved reliable in reducing dislocation rates in patients with femoral neck fractures, osteonecrosis of the femoral head, dysplasia, and rheumatoid arthritis, with reported aseptic-loosening rates of 0–0.5% [29,30,31,32].

Acetabular bone remodelling has been studied extensively in THA using standard highly porous cups. DEXA studies have shown an initial decline in periacetabular BMD during the first six months, followed by stabilization or modest recovery [33]. Retrieval studies have demonstrated bone ingrowth in 3D-printed acetabular components [34]. Other studies reported less periacetabular BMD reduction around highly porous 3D-printed cups than around hydroxyapatite-coated cups at longer follow-up [5], while short-term DEXA studies found no significant differences [9,35,36]. These findings provide biological context but cannot be directly extrapolated to the present comparison, which used radiographic RBD rather than DEXA or histological assessment.

The literature on periacetabular bone remodelling in THA with conventional DMCs remains limited. Tabori-Jensen et al. compared periacetabular bone remodelling between cemented and cementless DMCs of the same design [37]. They observed greater bone loss in the most cranioproximal region, increased BMD medially, and a smaller reduction in BMD below the cementless cup.

In our cohort, RLLs at final follow-up occurred only in the Conventional group. Radiographic filling adjacent to the superolateral rim was also observed in the Titanium group. Although the randomized trabecular titanium architecture is intended to support bone apposition and ingrowth [38], standard radiographs cannot distinguish bone ongrowth from ingrowth or provide histological confirmation. The disappearance of postoperative equatorial gaps may reflect bone filling, but over-reaming and biological ingrowth remain hypotheses. Moreover, an initial low-density gap adjacent to the cup may have influenced postoperative ROI 1 grayscale measurements. Previous studies of the same implant reported early radiographic signs consistent with fixation [39,40], whereas conventional DMCs have also shown satisfactory mid- to long-term radiographic performance [26,27,41,42]. Therefore, the current findings do not demonstrate superior fixation of the titanium design.

This study has several limitations. First, its retrospective comparative design is susceptible to selection bias and residual confounding. PSM included only age, sex, diagnosis and follow-up because other relevant factors—such as BMI, osteoporosis, smoking, diabetes, medication use, activity level, previous hip surgery and cup size—were not systematically available. Second, the cohort predominantly comprised older patients treated for femoral neck fracture, limiting generalizability to younger patients and elective osteoarthritis populations. Third, the Conventional group included several implant designs from different manufacturers, and the exact numerical distribution could not be reliably reconstructed despite an additional review of the available records. Differences in geometry, material, coating, fixation and stiffness may have increased heterogeneity. Moreover, because the relative contribution of each conventional design is unknown, one or more cup models may have disproportionately influenced the group-level results. This limits the interpretability of the Conventional group as a whole and prevents attribution of any finding to material or to a specific implant design. Fourth, validated clinical scores and patient-reported outcomes were not collected systematically. Fifth, the radiographs were not obtained using a study-specific acquisition protocol. Grayscale-derived RBD is less sensitive and less validated than DEXA, quantitative CT or RSA and is susceptible to positioning, pelvic rotation, beam alignment, exposure, scatter and post-processing. No direct comparison with DEXA was performed in this cohort. In addition, use of the prosthetic femoral head as the normalization reference may introduce bias because head material and size were not consistently available. Although both observers measured RBD independently, formal inter-observer ICCs were not calculated, and intra-observer reliability was unavailable because measurements were performed once. Sixth, Moore’s criteria are subjective, were developed for earlier porous-coated technologies and may not detect subtle differences between modern highly porous implants. Complete blinding to implant design could not be guaranteed despite image anonymization. Seventh, no a priori sample-size calculation was performed. The sensitivity analysis showed that the study could detect between-group Δ% RBD differences of approximately 6.2–8.6 percentage points, depending on the ROI; therefore, smaller but potentially clinically relevant differences may have remained undetected, and a type II error cannot be excluded. Multiple exploratory comparisons were performed without multiplicity correction, so the nominal RLL *p* value should be interpreted cautiously. Finally, the minimum 2-year follow-up permits assessment of early radiographic behaviour but cannot establish long-term remodelling, fixation, implant survival or revision risk.

## 5. Conclusions

At a minimum 2-year follow-up, the investigated 3D-printed titanium monobloc DMC demonstrated satisfactory radiographic fixation and no statistically significant differences in periacetabular RBD compared with a heterogeneous group of conventional DMCs. These findings indicate no detected short-term radiographic disadvantage but do not establish equivalence or biological superiority. Prospective, adequately powered studies incorporating validated clinical outcomes, repeated reliability assessment, quantitative imaging and longer follow-up are required.

## Figures and Tables

**Figure 1:**
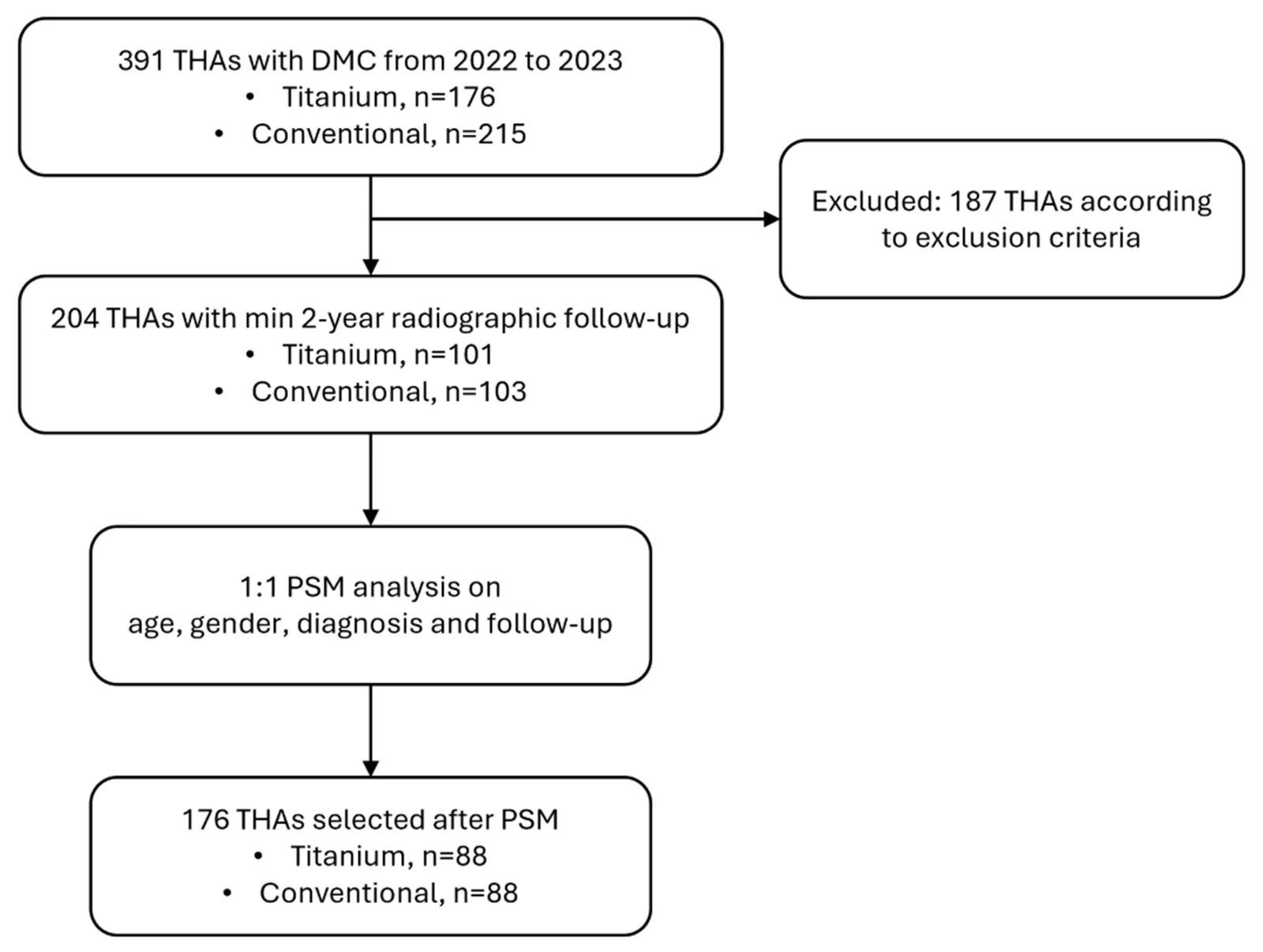
Flowchart of the study. From an initial cohort of 391 dual-mobility cups (DMCs), 187 were excluded. Following PSM for age, gender, diagnosis, and follow-up, 176 THAs (88 Titanium and 88 Conventional) were included in the final analysis.

**Figure 2:**
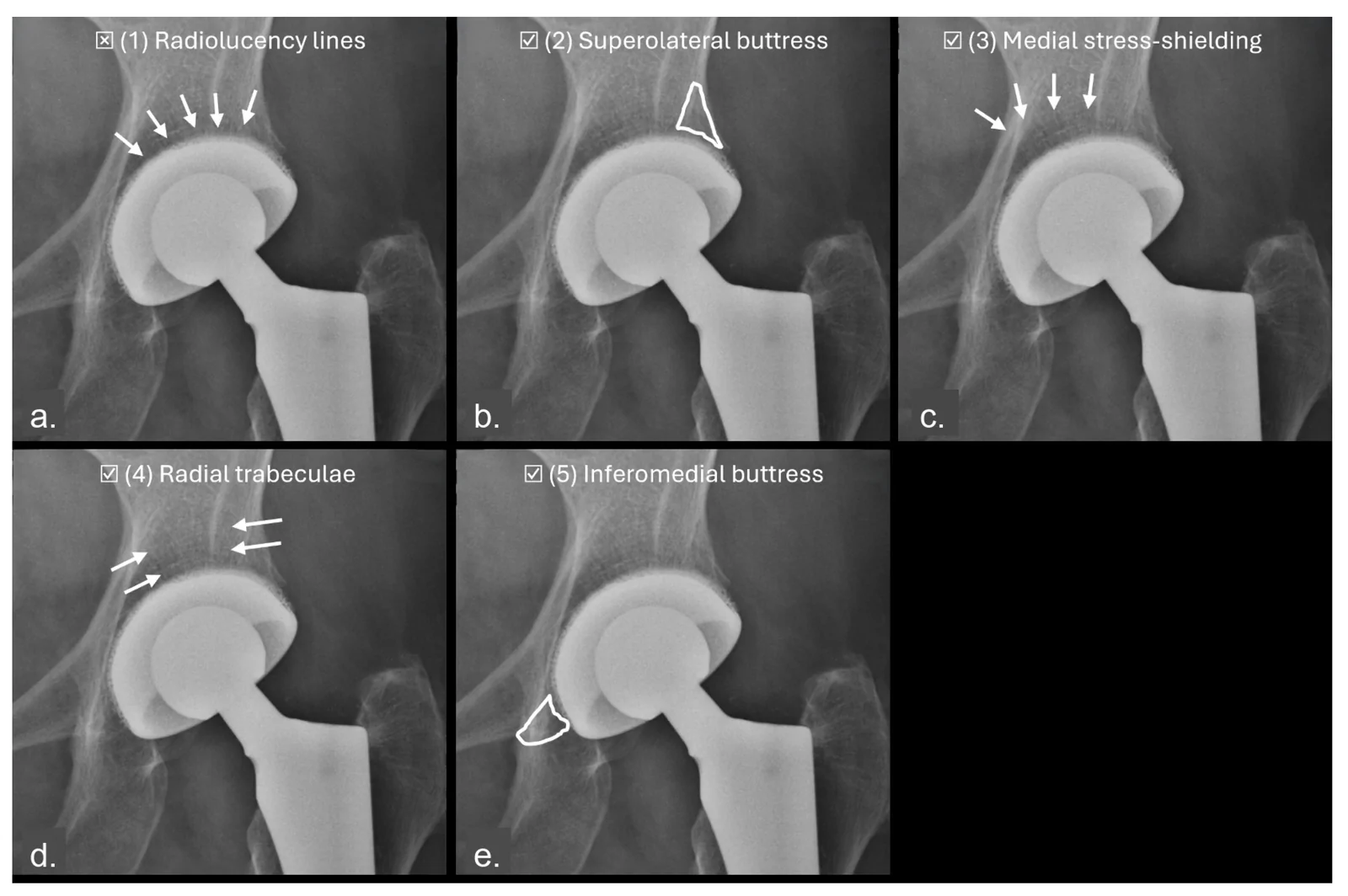
Radiographic evaluation methods. Moore’s signs of osseointegration are highlighted in white. (**a**) Radiolucent lines: arrows indicate the absence of gaps at the bone–implant interface. (**b**) Superolateral buttress: the outlined area highlights bone densification at the superolateral aspect of the acetabular cup. (**c**) Medial stress shielding: arrows indicate reduced bone density. (**d**) Radial trabeculae: arrows highlight the orientation of trabecular bone in response to the mechanical stress generated by the implant. (**e**) Inferomedial buttress: the outlined area indicates reactive bone formation at the inferomedial margin of the acetabulum.

**Figure 3:**
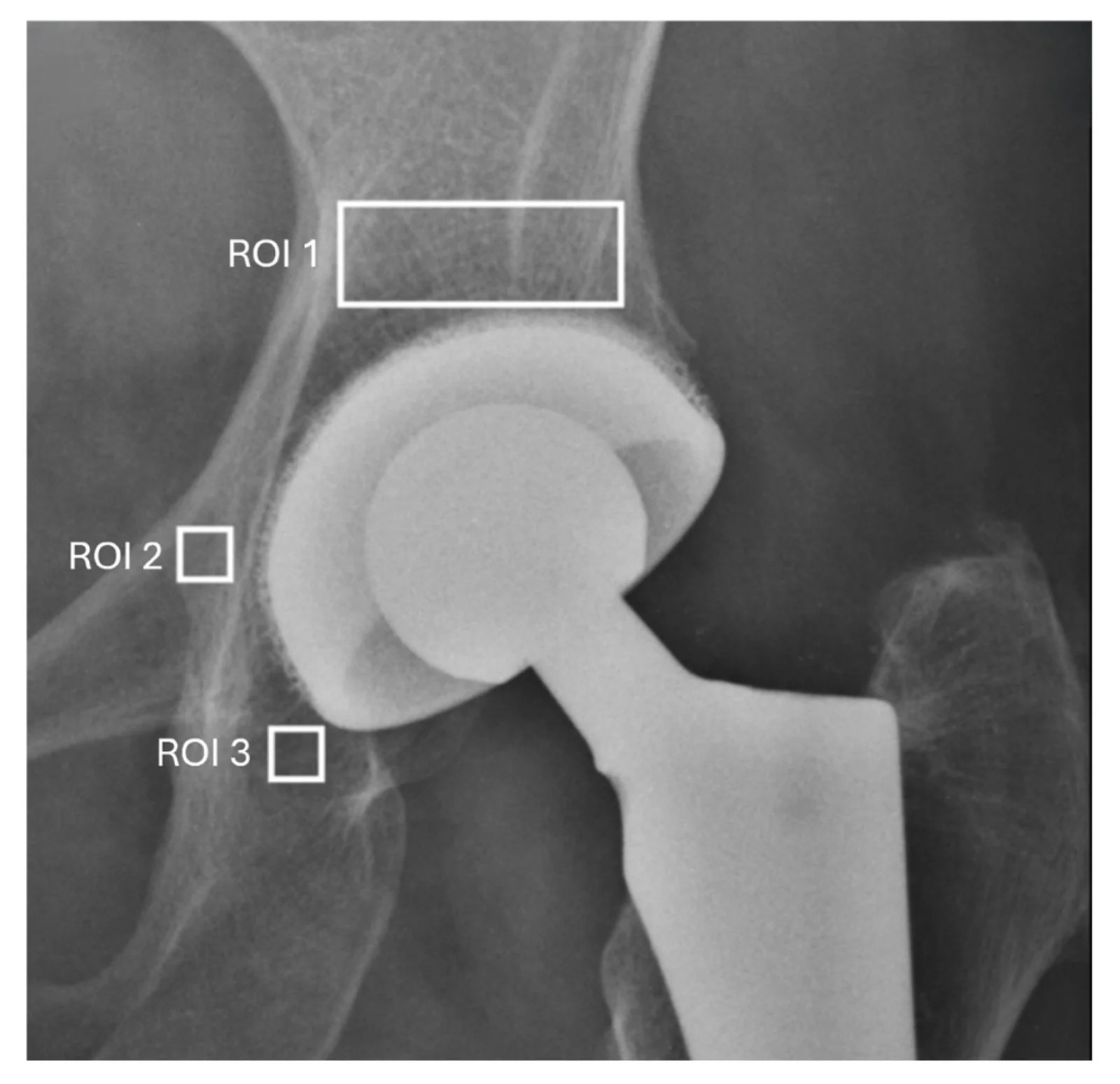
ROIs used for RBD analysis. The ROIs were selected to avoid superimposition of dense three-dimensional pelvic structures. ROI 1 was defined as a 10 mm high rectangle whose length depended on the width of the ilium. ROIs 2 and 3 were defined as 5 mm squares that did not cross the ilioischial line or include the teardrop.

**Figure 4:**
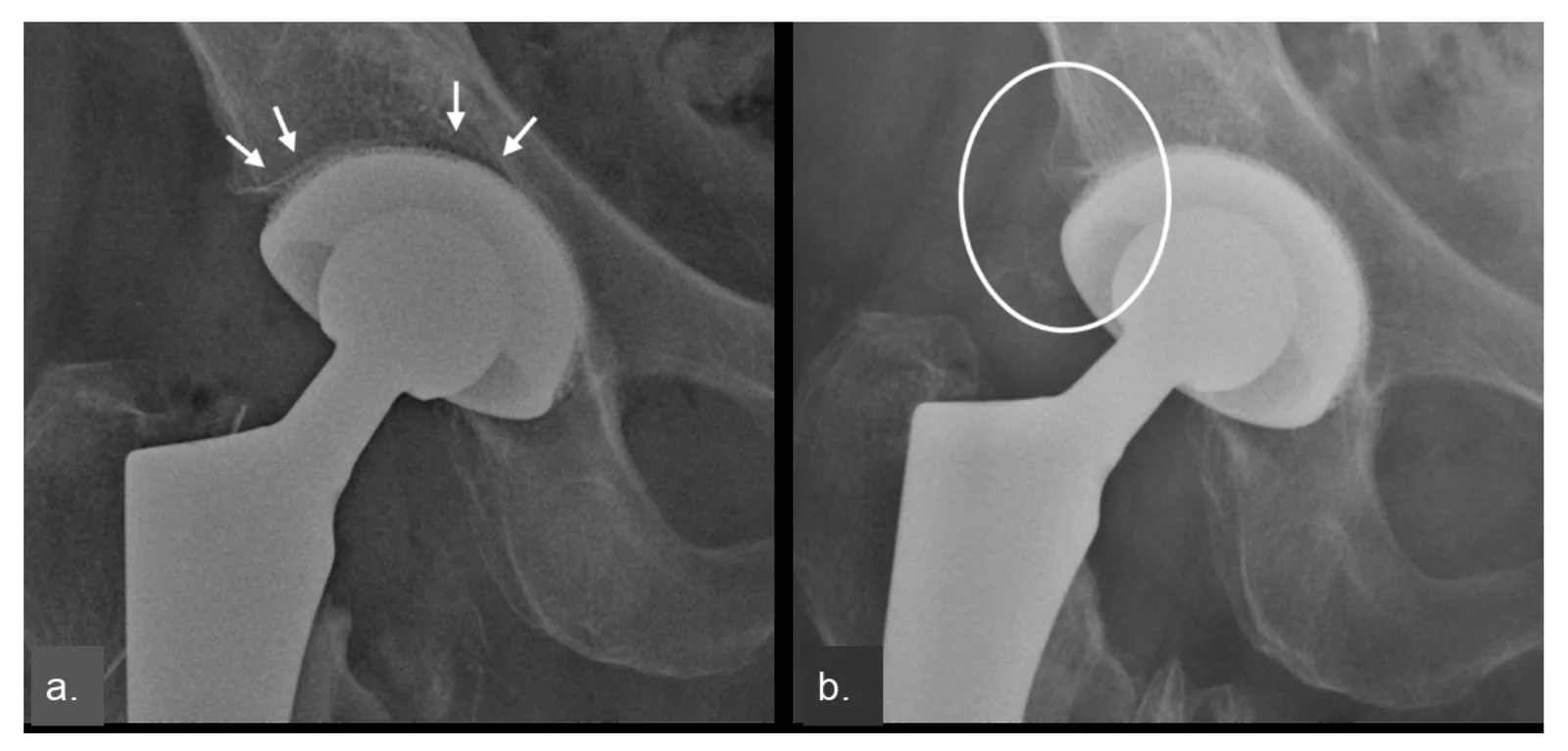
Radiographic signs of osseointegration with Titanium DMCs. (**a**) Postoperative and (**b**) follow-up radiographs of an 80-year-old woman who underwent surgery for a femoral neck fracture and received a titanium DMC. Postoperatively, the superolateral and polar reaming margins were visible (white arrows). At follow-up, new bone formation was evident in the superolateral and polar regions (white circle).

**Figure 5:**
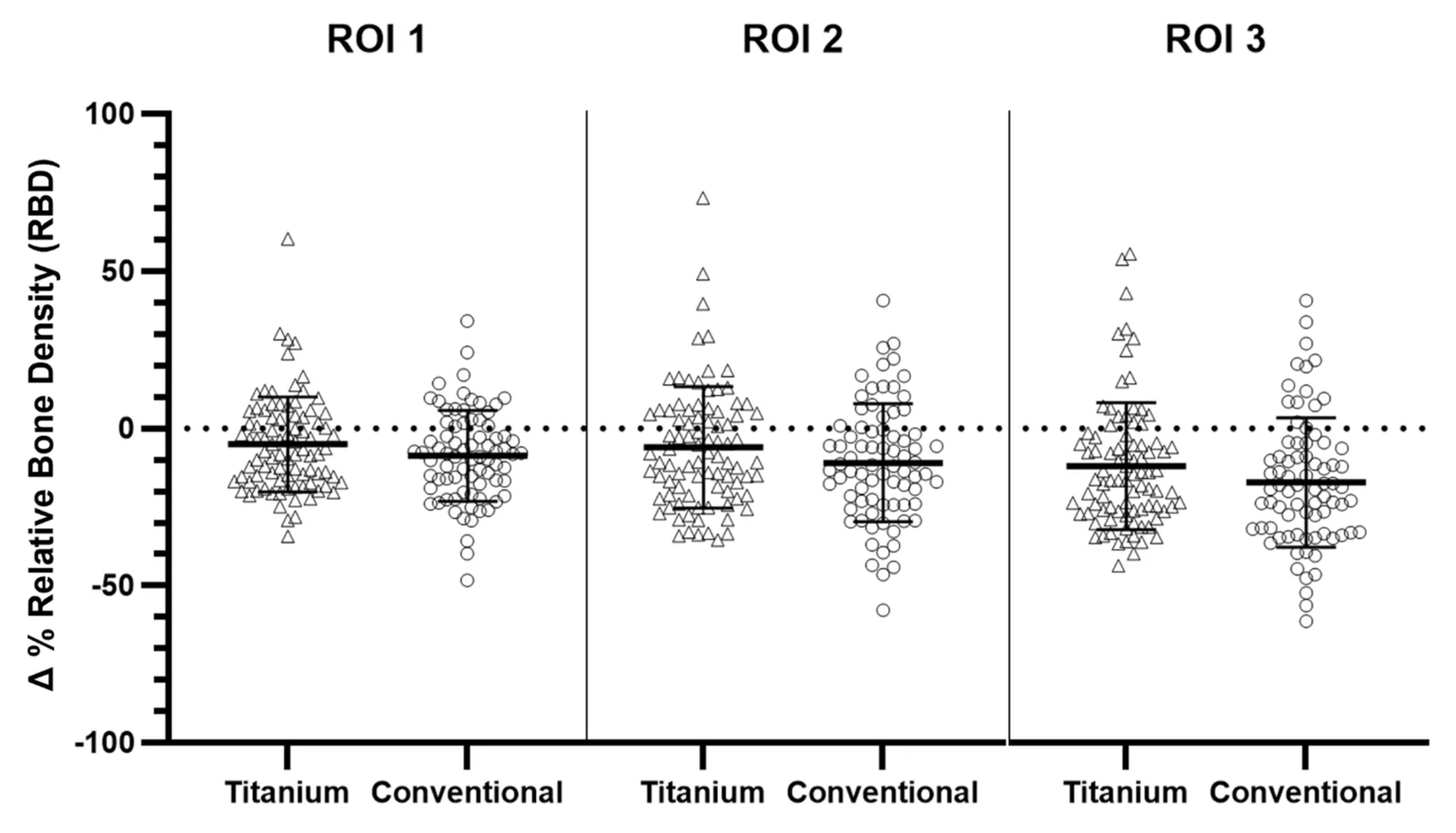
Comparison of RBD changes between the Titanium and Conventional groups. The scatter plots show the percentage change in RBD (Δ%) in the three ROIs around the cup. Data points for the Titanium group are represented by triangles (△), whereas those for the Conventional group are represented by circles (○). Horizontal bold lines indicate the mean for each group, and vertical error bars indicate the standard deviation.

**Table 1:** Patient characteristics before and after propensity score matching. (SD, standard deviation; FNF, femoral neck fracture; *p*, *p*-value; SMD, standardized mean difference; * *p* < 0.05).

		Before Propensity Score Matching				After Propensity Score Matching			
		Titanium	Conventional	*p*	SMD	Titanium	Conventional	*p*	SMD
No. of hips		101	103	-	-	88	88	-	-
Mean age (SD; range) [*years*]		75.5 (9.9; 38–89)	74.7 (10.4; 28–89)	0.624	0.077	75.4 (9.4; 40–87)	75.1 (10.7; 28–89)	0.815	0.028
Male:Female (%)		25:76 (24.8:75.2)	36:67 (34.9:65.1)	0.112	0.223	22:66 (25.0:75.0)	23:65 (26.1:73.9)	0.863	0.026
Diagnosis	FNF (%)	89 (88.1)	83 (80.6)	0.139	0.147	77 (87.5)	77 (87.5)	1.000	0.025
	Osteoarthritis (%)	9 (8.9)	17 (16.5)	0.089		9 (10.3)	9 (10.3)	1.000	
	Osteonecrosis (%)	2 (2.0)	2 (1.9)	1.000		2 (2.2)	1 (1.1)	1.000	
	Other (%)	1 (1.0)	1 (1.0)	1.000		0 (0.0)	1 (1.1)	1.000	
Mean Follow-up (SD; range) [*years*]		2.6 (0.4; 2.0–3.8)	2.4 (0.4; 2.0–3.7)	0.029 *	0.334	2.5 (0.4; 2.0–3.6)	2.5 (0.4; 2.0–3.7)	0.926	0.009

**Table 2:** Assessment of osseointegration signs and interobserver agreement. Frequencies (*n*) and percentages (%) are reported separately for each Moore sign, for the presence of at least three Moore signs, and for RLLs. (* *p* < 0.05).

	Titanium (88)				Conventional (88)				*p*
	Obs. 1 *n* (%)	Obs. 2 *n* (%)	κ	Agreem. *n* (%)	Obs. 1 *n* (%)	Obs. 2 *n* (%)	κ	Agreem. *n* (%)	
Moore 1	88 (100)	88 (100)	1.000	88 (100)	82 (93.2)	82 (93.2)	1.000	82 (93.2)	0.029 *
Moore 2	85 (96.6)	86 (97.7)	0.383	87 (98.9)	81 (92.0)	83 (94.3)	0.643	83 (94.3)	0.211
Moore 3	88 (100)	88 (100)	1.000	88 (100)	83 (94.3)	86 (97.7)	0.557	84 (95.4)	0.121
Moore 4	88 (100)	88 (100)	1.000	88 (100)	84 (95.4)	85 (96.6)	0.851	84 (95.4)	0.121
Moore 5	85 (96.6)	84 (95.4)	0.851	84 (95.4)	83 (94.3)	84 (95.4)	0.649	84 (95.4)	1.000
Sum ≥ 3	88 (100)	88 (100)	1.000	88 (100)	86 (97.7)	86 (97.7)	1.000	86 (97.7)	0.497
RLL	0	0	1.000	0	6 (6.8)	6 (6.8)	1.000	6 (6.8)	0.029 *

**Table 3:** Quantitative analysis of changes in RBD (Δ%). Means and standard deviations (SDs) are reported for each ROI, together with *p* values for comparisons between the Titanium and Conventional groups.

Δ% RBD (Mean ± SD)	Titanium	Conventional	*p*
ROI 1	−4.99 ± 15.11	−8.65 ± 14.39	0.218
ROI 2	−5.89 ± 19.30	−10.85 ± 18.68	0.160
ROI 3	−11.96 ± 20.15	−17.16 ± 20.59	0.138

## Data Availability

The raw data supporting the conclusions of this article will be made available by the authors on request.

## Abbreviations

The following abbreviations are used in this manuscript:

AP	Anterior–Posterior
BMD	Bone Mineral Density
CoCr	Cobalt–Chromium
DEXA	Dual-Energy X-ray Absorptiometry
DMC	Dual-Mobility Cup
PACS	Picture Archiving and Communication System
PSM	Propensity Score Matching
RBD	Relative Bone Density
RLL	Radiolucent Line
ROI	Region of Interest
SMD	Standardized Mean Difference
SS	Stainless Steel
THA	Total Hip Arthroplasty
